# Targeted PI3K/AKT/mTOR therapy for metastatic carcinomas of the cervix: A phase I clinical experience

**DOI:** 10.18632/oncotarget.2584

**Published:** 2014-10-29

**Authors:** Ming-Mo Hou, Xiaochun Liu, Jennifer Wheler, Aung Naing, David Hong, Robert L. Coleman, Apostolia Tsimberidou, Filip Janku, Ralph Zinner, Karen Lu, Razelle Kurzrock, Siqing Fu

**Affiliations:** ^1^ Department of Investigational Cancer Therapeutics, The University of Texas MD Anderson Cancer Center, Houston, Texas; ^2^ Department of Gynecologic Oncology, The University of Texas MD Anderson Cancer Center, Houston, Texas; ^3^ UC San Diego Moores Cancer Center, La Jolla, California; ^4^ Division of Hematology-Oncology, Chang Gung Memorial Hospital and Chang Gung University, Taoyuan, Taiwan

**Keywords:** Cervical Cancer, Phase I Trial, Matched Therapy, *PIK3CA* mutation, PTEN loss

## Abstract

**Background:**

Activated PI3K/AKT/mTOR pathway frequently occurs in metastatic or recurrent cervical carcinomas. However, the clinical benefits of matched therapy, a therapeutic approach targeting a specific mutational abnormality, have not yet been established.

**Methods:**

We analyzed the outcomes of patients with metastatic or recurrent cervical carcinomas who had a test for *PIK3CA* mutation and/or *PTEN* loss/mutation, and received ≥1 phase I therapeutic regimen between January 2006 and June 2013.

**Results:**

Patients with adenocarcinoma had fewer *PIK3CA* mutations (14%), and survived longer (median, 14.2 months) than those with squamous cell carcinoma (48% and 7.2 months; p = 0.016, and 0.001, respectively). Matched therapy targeting the activated PI3K/AKT/mTOR pathway led to a favorable rate of SD ≥ 6 months/CR/PR (53%) and significantly longer progression-free survival (median, 6.0 months) than non-matched therapy (11% and 1.5 months; p = 0.08 and 0.026; respectively). In patients with squamous cell carcinoma of the cervix, the presence of *PIK3CA* mutations was associated with a significantly longer overall survival (median, 9.4 months) than the absence of *PIK3CA* mutations (median, 4.2 months; p = 0.019).

**Conclusions:**

Matched therapy targeting the activated PI3K/AKT/mTOR pathway provided meaningful clinical benefits. Thus, further evaluation of PI3K/AKT/mTOR pathway targeted therapy is warranted, especially in metastatic or recurrent squamous cell carcinoma.

## INTRODUCTION

Cancer of the uterine cervix is one of the most common gynecologic malignancies and causes of death worldwide [1]. In the United States, more than 12,000 women were diagnosed with cervical cancer in 2013, and more than 4,000 patients died from this disease [2]. Squamous cell carcinoma and adenocarcinoma account for about 95% of all cervical cancers [3]. Most patients who are diagnosed with early-stage disease have a high rate of long-term survival after they have undergone curative surgical resection and/or chemoradiotherapy [4, 5]. Patients with recurrent or metastatic cervical cancers who are not amenable to radical local excision or curative chemoradiotherapy, have a poor prognosis [6, 7]. Palliative systemic therapies such as platinum-based doublets plus an anti-angiogenic agent as the standard of care for first-line treatment yield modest survival gains of 3.7 months [8–10]. Subsequent conventional chemotherapeutic regimens result in increased toxicity with limited clinical benefit [11]. The overall poor prognosis of these patients warrants the urgency for the development of novel therapeutic regimens [12, 13].

There are three classes of phosphoinositide 3-kinase (PI3K) isoforms [14]. Class I PI3Ks are heterodimeric lipid kinases composed of the p110 catalytic subunit and the p85 regulatory subunit. Three major p110 isoforms have been described: p110α (*PIK3CA*), p110β (*PIK3CB*), and p110δ (*PIK3CD*) [15, 16]. PI3K and PTEN counterpoise each other on conversion between phosphatidyl-inositol-4-5-bisphosphate (PIP2) and phosphatidyl-inositol-3, 4, 5-trisphosphate (PIP3) [17, 18]. Upon activation through either *PIK3CA* mutation or *PTEN* loss/mutation, PI3K phosphorylates PIP2 to PIP3, which facilitates recruitment and activation of AKT to initiate a cascade of downstream signaling events including the mTOR complex, a major downstream pathway [16]. Therefore, a regimen including a PI3K inhibitor and/or an mTOR inhibitor can be utilized to target *PIK3CA* mutation and/or *PTEN* loss/mutation-mediated activated PI3K/AKT/mTOR pathway [19]. This strategy is defined as matched therapy: a therapeutic regimen including an agent (either as a single agent or as a part of a combination regimen) that is known to directly inhibit a specific mutation, and/or to inhibit its key downstream pathways, such as treatment with a PI3K inhibitor, an mTOR inhibitor, or these agent-based regimens for a cancer patient carrying a *PIK3CA* mutation and/or *PTEN* loss/mutation [20].

Our previous study and others have documented that the activated PI3K/AKT/mTOR pathway is frequently identified in patients with metastatic or recurrent cervical cancers [21–25]. We hypothesized that cervix cancer patients with aberrations in this pathway would achieve clinical benefit (defined as objective response and prolonged stable disease) when treated with PI3K/AKT/mTOR pathway targeted agents. In this article, we further our previous study by limiting patients who only have either squamous cell carcinoma or adenocarcinoma of the cervix in order to reach definitive conclusions, which will be applied to facilitate future drug development by choosing appropriate therapeutic regimens for appropriate cancer patients. Herein we document a clear observation of antitumor responses with acceptable toxicity from PI3K/AKT/mTOR matched therapy, representing an important new therapeutic strategy for a patient population possessing limited treatment options.

## PATIENTS AND METHODS

### Patient selection

Consecutive patients with metastatic or recurrent cervical carcinoma (either squamous cell carcinoma or adenocarcinoma) who were referred to the Department of Investigational Cancer Therapeutics (A Phase I Clinical Trials Program) at MD Anderson between January 1, 2006, and June 30, 2013, had a test for *PIK3CA* mutation and/or *PTEN* loss/mutation in a Clinical Laboratory Improvement Amendments-certified molecular diagnostic laboratory, and received treatment in at least one phase I clinical trial, were evaluated. Follow-up was defined as the time of the initial office visit after the patient was referred to the phase I clinic until death or February 28, 2014. This study was conducted in accordance with MD Anderson's Institutional Review Board guidelines.

### Data collection

Two individuals independently reviewed patients' electronic medical records at MD Anderson and crosschecked the collected data. Clinical information that was collected included race, prior treatment history (e.g., surgery, radiation therapy, and chemotherapy), date of birth, Eastern Cooperative Oncology Group performance status at the initial phase I clinic visit, mutation profiling of the tumor specimen including *PIK3CA* mutation and/or *PTEN* loss/mutation status, phase I clinical trial therapies, and clinical outcomes: severe adverse event (SAEs), progression-free survival (PFS), overall survival (OS), and objective responses including complete remission (CR), partial response (PR), and stable disease for 6 months or longer (SD ≥ 6months).

SAEs were defined as toxic effects that were grade 3 or higher according to the National Cancer Institute Common Terminology Criteria for Adverse Events v3.0 or v4.0 (http://ctep.cancer.gov/reporting/ctc.html) [26]. Clinical objective responses were evaluated according to the Response Evaluation Criteria in Solid Tumors version 1.0 or 1.1 per individual study protocols [27, 28]. PFS was defined as the interval from the date of initial treatment to the first objective documentation of disease progression, the time of death if it occurred, or the last date of contact on February 28, 2014, at which time the patients' data were censored. OS was estimated from the date of the initial phase I clinical trial therapy to death or the last date of contact on February 28, 2014, at which time the patients' data were censored.

Enrollment of an eligible patient into a specific phase I trial depended on the availability of the phase I trial at the time of presentation and the preference of the treating physician according to good clinical practice. If a phase I trial was unsuccessful, another available phase I trial was introduced as long as a patient was eligible and willing to participate.

### Statistical analyses

Categorical data were described using contingency tables. Continuously scaled measures were summarized with descriptive statistical measures (i.e., the median with the range), whereas PFS and OS rates were estimated using the Kaplan-Meier method. Patients who were still alive at the time of data analysis were censored at that time. Fisher's exact test was used to assess the associations between categorical variables and mutation status. Statistical inferences were based on two-sided tests at a significance level of *p* < 0.05. Statistical analyses were carried out using SPSS Statistics version 22 (IBM Inc., Armonk, NY).

## RESULTS

### Study population

Fifty-five consecutive patients with metastatic or recurrent adenocarcinoma (n = 24) or squamous cell carcinoma (n = 31) who had a test for *PIK3CA* mutation and/or *PTEN* loss/mutation and underwent treatment in at least one phase I clinical trial in the Phase I Clinical Trials Program were included in this study. Patient characteristics and molecular profiling are listed in Table 1. All patients had received at least one prior systemic chemotherapeutic regimen for metastatic or recurrent disease (median 2 regimens, range 1 to 4) and underwent molecular marker studies in a Clinical Laboratory Improvement Amendments-certified molecular diagnostic laboratory.

**Table 1:** Patient characteristics and molecular profiles

Patient characteristics	Adenocarcinoma	Squamous cell carcinoma	Total patients
	(n = 24)	(n = 31)	(n = 55)
**Age, years**			
Median (range)	51 (27–67)	42 (25–69)	46 (25–69)
**Race**			
White	17	19	36
African American	2	5	7
Hispanic	3	6	9
Others	2	1	3
**Prior therapies**			
Surgery	15	12	27
Radiotherapy	23	28	51
Chemotherapy	24	31	55
Antiangiogenic	3	6	9
Median regimen	2	2	2
(range)	(1–4)	(1–4)	(1–4)
**Eastern cooperative oncology group performance status**			
0	8	7	15
1	14	17	31
2	2	7	9
**Molecular profiles**		Aberrant/tested (%)	
*PIK3CA*	3/22 (14)	14/29 (48)	17/51 (33)
*PTEN*	4/16 (25)	3/24 (13)	7/40 (18)
*TP53*	3/7 (43)	1/12 (8)	4/19 (21)
*HER2*	1/9 (11)	1/14 (7)	2/23 (9)
*CKIT*	0/11 (0)	2/18 (11)	2/29 (7)
*CMET*	0/11 (0)	2/18 (11)	2/29 (7)
*KRAS*	3/19 (16)	0/26 (0)	3/45 (7)
*ALK1*	0/7 (0)	1/13 (8)	1/20 (5)
*EGFR*	1/16 (6)	0/22 (0)	1/38 (3)
*BRAF*	0/18 (0)	0/25 (0)	0/43 (0)
*NRAS*	0/13 (0)	0/18 (0)	0/31 (0)
*AKT1*	0/8 (0)	0/14 (0)	0/22 (0)

Twenty-two patients were found to harbor *PIK3CA* mutations and/or PTEN loss: 17 (33%) of 51 tested patients with *PIK3CA* aberrations (E545K = 8, E542K = 4, H1047L = 1, H1047R = 1, E545K and D549H = 1, E542K and M1043I = 1, amplification = 1); 7 (18%) of 40 tested patients with PTEN loss (n = 6) and/or mutation (R173C = 1); and 2 patients have both of PIK3CA mutation (E545K) and PTEN loss. Further analyses revealed that patients with squamous cell carcinomas were significantly younger and more likely to carry *PIK3CA* mutations than those with adenocarcinomas (*p* = 0.034 per independent samples t-test for age and p = 0.016 per Fisher's exact test for *PIK3CA* mutations, respectively). Approximately 20% (n = 11/55) and 36% (n = 4/11) of the referred patients received subsequent second- and third-line phase I trial therapies.

### Severe adverse events

All patients were included for analyses of SAEs; a total of 450 cycles of therapy were administered according to 38 phase I clinical trials [21]. During their first phase I trial therapy, approximately 20% of patients (n = 11/55) experienced 19 episodes of SAEs: neutropenia and thrombocytopenia (n = 5 each, 9%); hyponatremia (n = 2, 4%); and hypokalemia, hypomagnesaemia, hypophosphatemia, mucositis, fatigue, bowel perforation, and bacterial infection (n = 1 each, 2%). During the second phase I trial therapy, only 1 (9%) of 11 patients had an SAE (fatigue). Subsequently, no patient experienced an SAE before all 4 patients progressed within the initial 8 weeks of their phase I trial therapy. In patients who received PI3K/AKT/mTOR pathway targeted therapeutic regimens, no apparent difference was observed between patients with *PI3KCA* mutation and/or *PTEN* loss/mutation and those without.

### Antitumor activity

**Figure 1:**
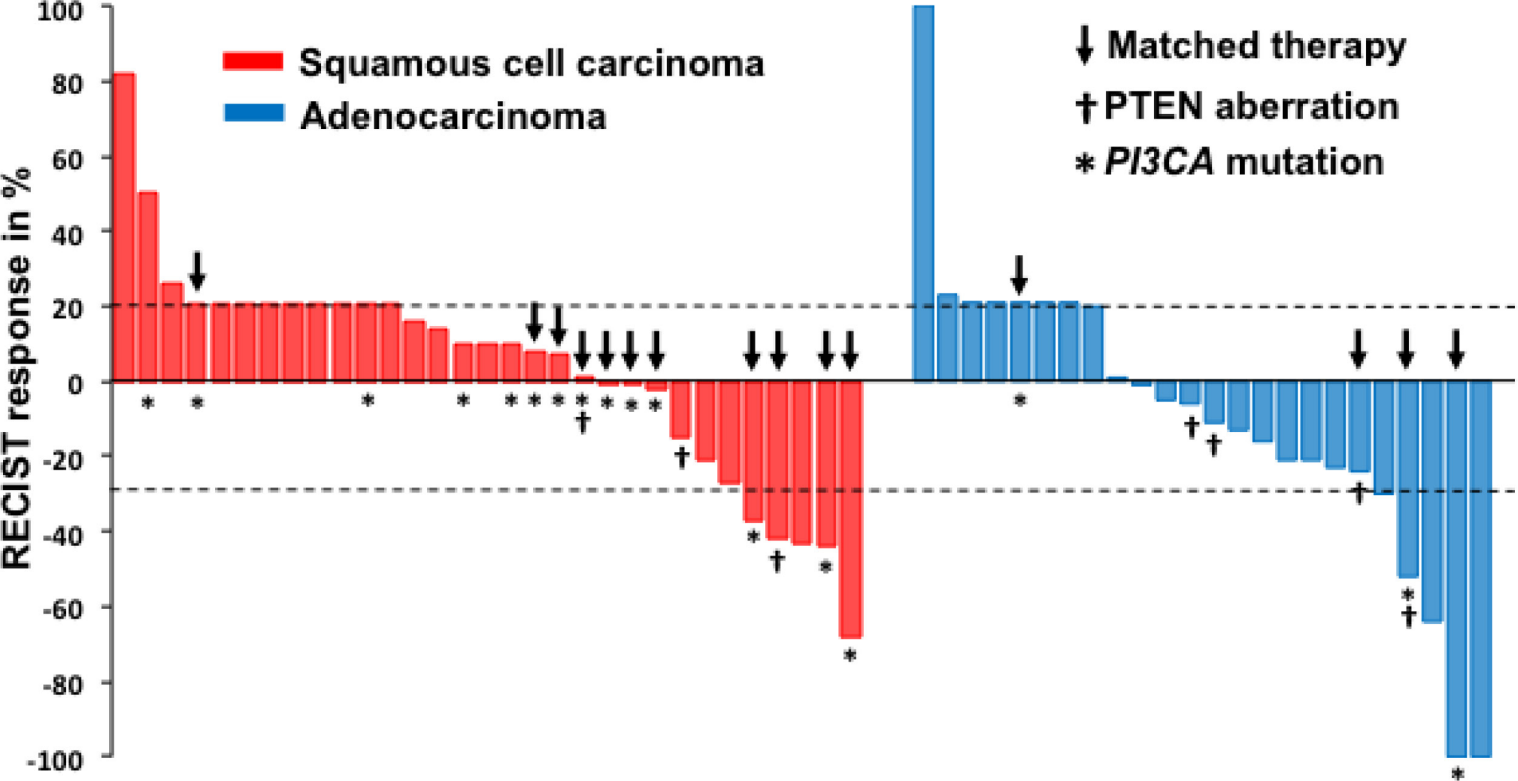
The waterfall plot shows the best tumor response to their initial phase I clinical trial therapy (n = 55)

**Figure 2:**
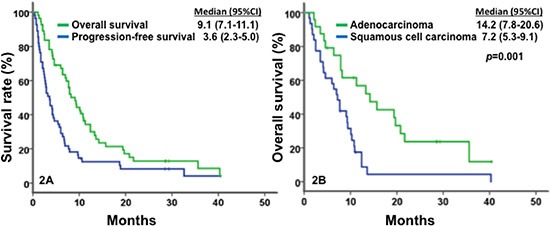
Kaplan-Meier plots for survivals In Figure 2A, a median OS of 9.1 months (95% CI, 7.1–11.1, in green) and a median PFS of 3.6 months (95% CI, 2.3–5.0, in blue) were observed in patients with metastatic or recurrent cervical carcinomas (n = 55). In Figure 2B, patients with adenocarcinoma (in green) showed a median OS of 14.2 months (n = 24; 95% CI, 7.8–20.6), significantly longer those with squamous cell carcinoma (in blue), 7.2 months (n = 31; 95% CI, 5.3–9.1; *p* = 0.001).

**Figure 3:**
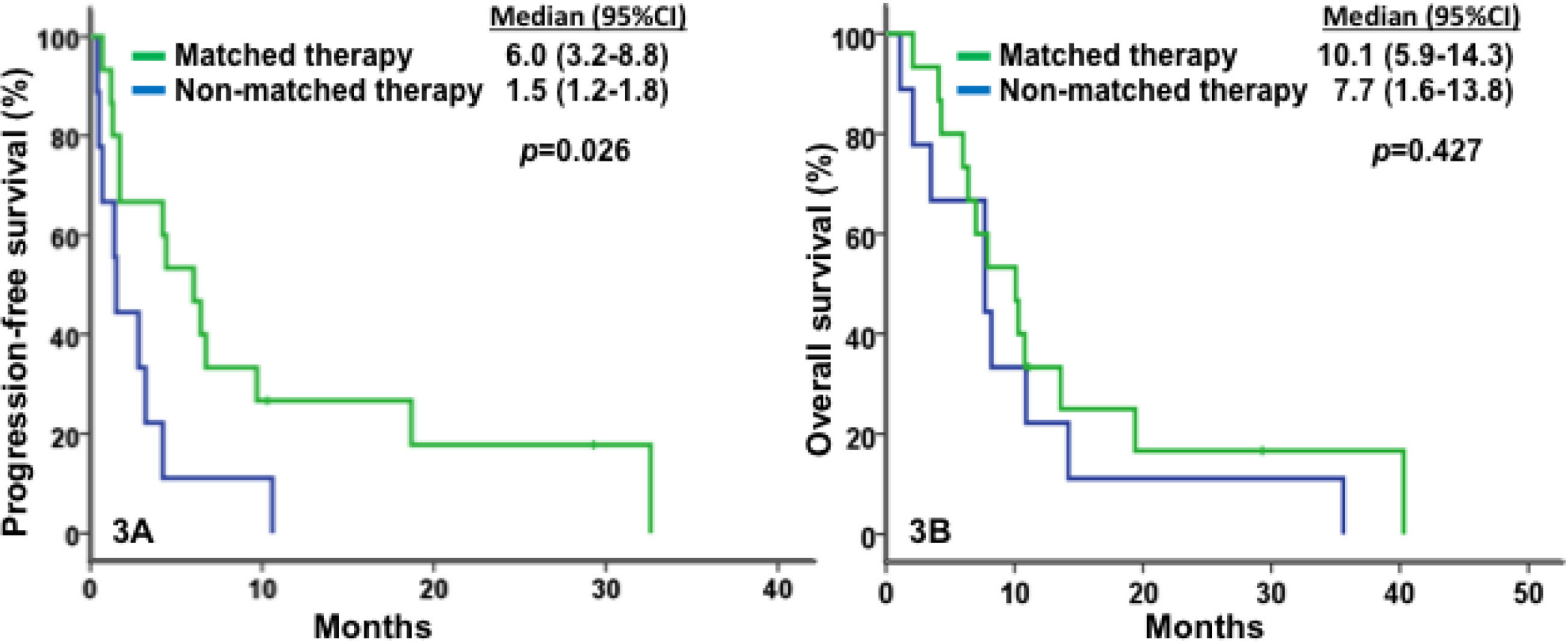
Kaplan-Meier plots for survivals In Figure 3A, match therapy (in green) was associated with a median PFS of 6.0 months (n = 15; 95% CI, 3.2–8.8), significantly greater than non-matched therapy (in blue) with 1.5 months (n = 9; 95% CI, 1.2–1.8; *p* = 0.026). In Figure 3B, matched therapy (in green) was associated with a median OS of 10.1 months (n = 15; 95% CI, 5.9–14.3), compared to non-matched therapy (in blue) with 7.7 months (n = 9; 95% CI, 1.6–13.8; *p* = 0.427).

The waterfall plot shown in Figure 1 demonstrates the best tumor response to their first-line phase I clinical trial therapy. Of 55 patients who received their first-line phase I trial therapy (see Tables 2 and 3), 35% achieved SD ≥ 6 months/CR/PR (CR = 2, PR = 8, and SD ≥ 6 months = 9), and the median PFS in this group was 3.6 months (95% confidence interval [CI], 2.3–5; Figure 2A). Of 15 patients with *PIK3CA* mutations and/or *PTEN* loss/mutations who received matched therapy, 53% achieved SD ≥ 6 months/CR/PR (CR = 1, PR = 5 and SD ≥ 6 months = 2), and the median PFS in this group was 6.0 months (95% CI, 3.2–8.8). These results compared favorably with nine patients who did not receive matched therapy targeting activated PI3K/AKT/mTOR pathway: 11.1% achieved PR (PR = 1; *p* = 0.08), and the median PFS in this group was 1.5 months (95% CI, 1.2–1.8; *p* = 0.026; Figure 3A); as well as with seven patients without *PIK3CA* mutation and *PTEN* loss/mutations who received therapies targeting activated PI3K/AKT/mTOR pathway: 14% achieved PR (PR = 1, p = 0.16) and the median PFS in this group was 1.5 months (95% CI, 0.4 – 10.6; p = 0.06).

**Table 2:** Clinical outcomes in patients with advanced squamous cell carcinoma of the cervix

Age	PIK3CA mutation	PTEN loss or mutation	Best tumor response	PFS (months)	OS (months)	Matched therapy	Treatment
35	E542K	ND	−44%	9.7	10.1	Y	Bevacizumab and Temsirolimus plus Doxil
37	E542K	N	−37%	10.3	10.3	Y	A PI3K Inhibitor
37	E542K	N	−2%	4.2	6.4	Y	Everolimus and Pazopanib
42	E542K	N	21%	0.9	2.5	N	Erlotinib and Praletrexate
59	E542K / M1043I	N	50%	1.7	7.8	N	Erlotinib and Praletrexate
35	E545K	Y	0%	4.4	10.8	Y	Bevacizumab and Temsirolimus plus Doxil
56	E545K	P	8%	6.4	13.6	Y	Sirolimus and Docetaxol
48	E545K	ND	−68%	32.6	40.3	Y	Bevacizumab and Temsirolimus
40	E545K	ND	7%	1.2	4.1	Y	Bevacizumab and Temsirolimus plus Doxil
38	E545K	N	−1%	6.7	7.0	Y	Bevacizumab and Temsirolimus plus Doxil
46	E545K	N	10%	5.4	9.3	N	Lenalidomide and Bevacizumab
48	E545K	N	10%	3.5	9.4	N	HAI Abraxane and IV Gemcitabine plus Bevacizumab
62	E545K / D549H	ND	21%	0.7	2.1	Y	Bevacizumab and Temsirolimus plus Doxil
61	H1047R	N	21%	1.3	1.3	Y	A PI3K Inhibitor
28	N	Y	−42%	6.0	6.0	Y	Bevacizumab and Temsirolimus plus Doxil
49	N	Y	−15%	2.6	3.5	N	Bendamustine and Bevacizumab
62	N	P	21%	2.4	2.5	N	HAI Abraxane
36	N	P	82%	1.5	7.7	N	Bevacizumab and Temsirolimus plus Doxil
39	N	P	21%	1.1	1.3	N	A MEK Inhibitor and Docetaxol
41	N	P	21%	0.4	1.1	N	Bevacizumab and Temsirolimus plus Carboplatin
34	N	ND	−43%	6.8	9.1	N	Bevacizumab and Temsirolimus
25	N	ND	14%	4.2	7.7	?	A PI3K Inhibitor and Carboplatin plus Paclitaxel
37	N	ND	−27%	1.4	10.9	?	Bevacizumab and Temsirolimus plus Doxil
43	N	N	10%	7.9	12.4	N	Erlotinib and Praletrexate
58	N	N	16%	4.1	4.6	N	Erlotinib and Praletrexate
29	N	N	21%	2.7	4.2	N	Bendamustine and Bevacizumab
69	N	N	21%	1.2	1.7	N	Erlotinib and Praletrexate
45	N	N	21%	0.9	1.8	N	HAI Oxaliplatin and PO Capecitabine
63	N	N	21%	0.5	0.7	N	Everolimus and Denosumab
48	ND	P	−21%	6.9	12.4	?	Bevacizumab and Temsirolimus plus Doxil
37	ND	N	26%	2.3	7.2	N	Trientine and Carboplatin

I, insufficient; N, no; ND, not done; OS, overall survival; P, partial loss; PFS, progression-free survival; Y, yes; +, censored; and ?, unknown.

**Table 3:** Clinical outcomes in patients with advanced adenocarcinoma of the cervix

Age	PIK3CA mutation	PTEN loss or mutation	Best tumor response	PFS (months)	OS (months)	Matched therapy	Treatment
55	Amplification	P	−100%	29.3 +	29.3 +	Y	Everolimus and Anastrozole
61	E545K	Y	−52%	18.7	19.4	Y	Bevacizumab and Temsirolimus
51	H1047L	N	21%	1.7	4.3	Y	Sirolimus and Metformin
63	N	Y	−24%	1.7	7.9	Y	Bevacizumab and Temsirolimus plus Doxil
41	N	Y	−11%	28.5 +	28.5 +	N	An Aurora Kinase Inhibitor
50	N	R173C	−6%	7.7	11.3	N	Pazopanib and Pemetrexate
44	N	P	−21%	9.8	15.7	N	A MEK Inhibitor and Docetaxol
62	N	P	20%	2.1	19.7	N	A Proteasome Inhibitor
43	N	P	−13%	40.4 +	40.4 +	N	A c-Met Inhibitor
51	N	ND	−21%	6.0	6.4	?	Bevacizumab and Temsirolimus
58	N	ND	0%	5.7	7.9	?	Bevacizumab and Temsirolimus plus Doxil
27	N	ND	−29%	3.7	21.7	?	Bevacizumab and Cetuximab plus Erlotinib
46	N	ND	−16%	2.9	3.5	?	Bevacizumab and Temsirolimus plus Doxil
58	N	ND	430%	2.5	20.8	?	Temsirolimus and Topotecan plus Bortezomib
63	N	ND	21%	2.1	2.4	N	Azacitidine and Oxaliplatin
44	N	N	−100%	18.9	28.7 +	N	An Aurora Kinase Inhibitor and Docetaxol
37	N	N	−64%	10.6	35.6	N	A PI3K Inhibitor and Carboplatin plus Paclitaxel
59	N	N	0%	3.7	5 +	N	Bevacizumab and VEGF Inhibition
57	N	N	−23%	3.2	14.2	N	Bevacizumab and Temsirolimus
49	N	N	−5%	2.9	4.6	N	An Aurora Kinase Inhibitor
41	N	N	23%	2.8	8.2	N	Bevacizumab and Temsirolimus plus Paclitaxel
60	N	N	21%	0.7	2.1	N	Bevacizumab and Temsirolimus plus Sorafenib
67	ND	I	21%	3.6	10.7 +	?	Everolimus and Anastrozole
40	ND	I	21%	1.1	13.3	?	Bevacizumab and Temsirolimus plus Doxil

I, insufficient; N, no; ND, not done; OS, overall survival; P, partial loss; PFS, progression-free survival; Y, yes; +, censored; and ?, unknown.

Of 11 patients who received their second phase I clinical trial therapy, 27% achieved SD ≥ 6 months/CR/PR (PR = 1 and SD ≥ 6 months = 2), and the median PFS in this group was 4.2 months (95% CI, 3.0–5.4). Of 4 patients who received their third phase I clinical trial therapy, no patient achieved SD ≥ 6 months/CR/PR.

### Overall survivals

**Figure 4:**
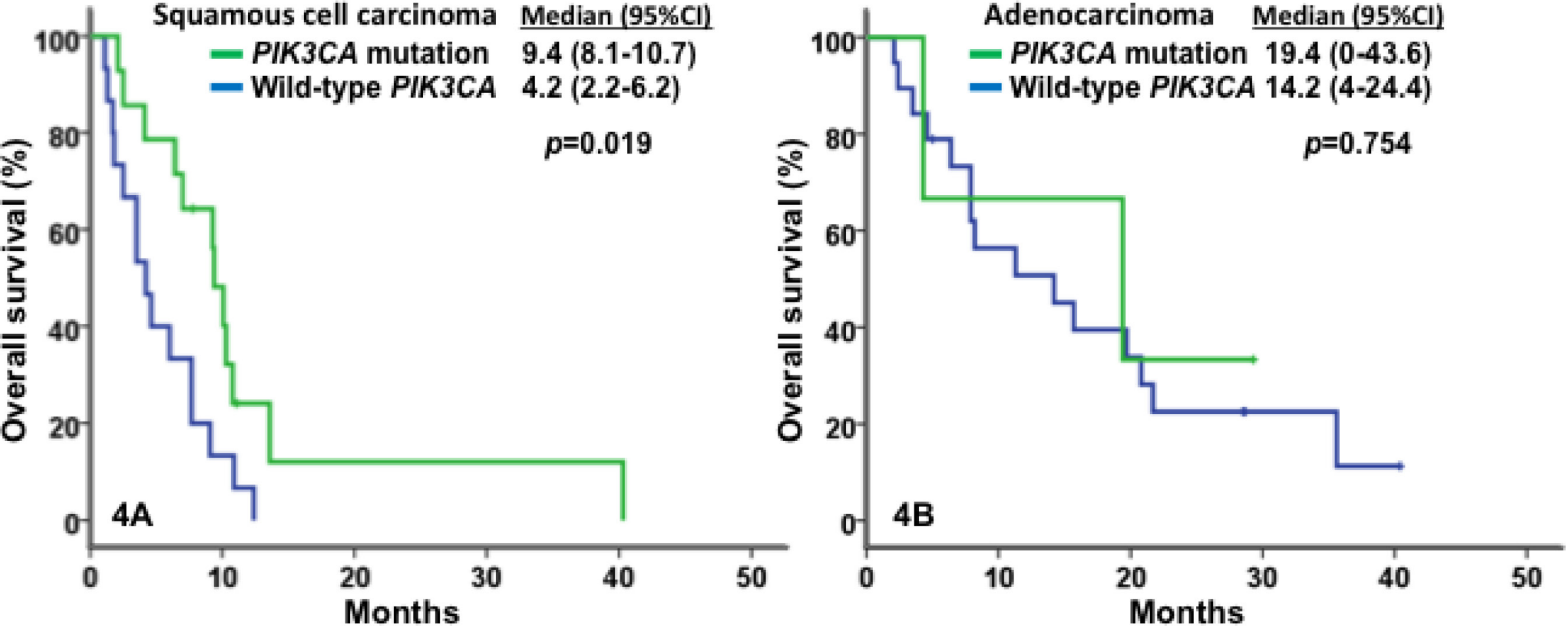
Kaplan-Meier plots for survivals In Figure 4A for in patients with squamous cell carcinoma, *PI3KCA* mutations (in green) were associated with a median OS of 9.4 months (n = 14; 95% CI, 8.1–10.7), significantly longer than wild-type *PI3KCA* (in blue) with 4.2 months (n = 15; 95% CI, 2.2–6.2; *p* = 0.019). In Figure 4B for patients with adenocarcinoma, *PI3KCA* mutations (in green) were associated with a median OS of 19.4 months (n = 3; 95% CI, 0–43.6), compared to wild-type *PI3KCA* (in blue) with 14.2 months (n = 19; 95% CI, 4–24.4; *p* = 0.754).

**Figure 5:**
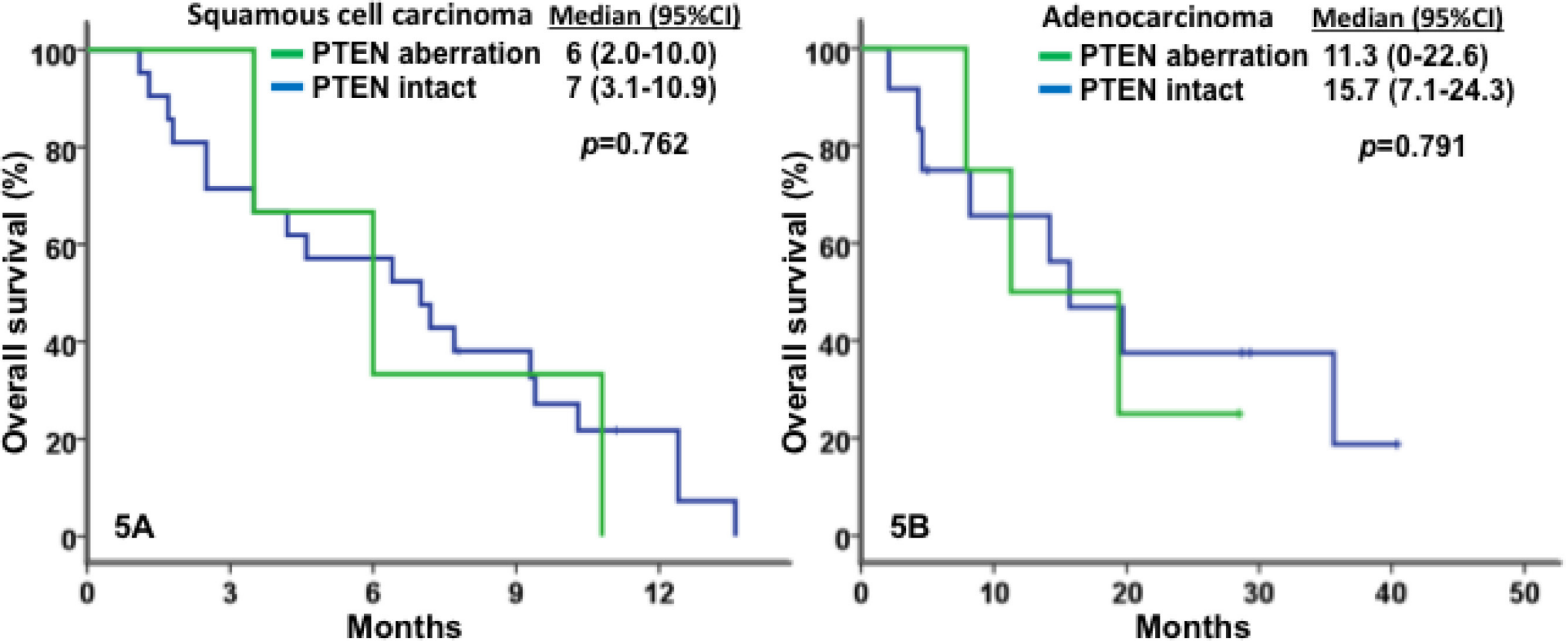
Kaplan-Meier plots for survivals In Figure 5A for in patients with squamous cell carcinoma, PTEN aberrations (in green) were associated with a median OS of 6 months (n=3; 95% CI, 2–10), similar to PTEN intact (in blue) with 7 months (n = 21; 95% CI, 3.1–10.9; *p* = 0.762). In Figure 5B for patients with adenocarcinoma, PTEN aberrations (in green) were associated with a median OS of 11.3 months (n = 4; 95% CI, 0–22.6), similar to PTEN intact (in blue) with 15.7 months (n = 16; 95% CI, 7.1–24.3; *p* = 0.791).

All patients were included in survival analyses. The median OS was 9.1 months (95% CI, 7.1–11.1 months; Figure 2A). Patients with metastatic or recurrent adenocarcinomas had a significantly greater median OS of 14.2 months (95% CI, 7.8–20.6) than those with squamous cell carcinomas (OS = 7.2 months; 95% CI, 5.3–9.1; *p* = 0.001), as shown in Figure 2B. Patients with *PIK3CA* mutation and/or *PTEN* loss/mutation who received matched therapy, achieved a median OS of 10.1 months (95% CI, 5.9–14.3), compared with those who did not (7.7 months; 95% CI, 1.6–13.8; p = 0.43; Figure 3B). Furthermore, patients with metastatic or recurrent squamous cell carcinomas who carried *PIK3CA* mutations (n = 14) achieved a median OS of 9.4 months (95% CI, 8.1–10.7), significantly longer than the median OS of those who did not carry *PIK3CA* mutations (n = 15; 4.2 months, 95% CI, 2.2–6.2; *p* = 0.019), as shown in Figure 4A. Patients with metastatic or recurrent adenocarcinomas who carried *PIK3CA* mutations (n = 3) achieved a median OS of 19.4 months (95% CI, 0 – 43.6), similar to those who did not carry *PIK3CA* mutations (n = 19; 14.2 months, 95% CI, 4.0–24.4; *p* = 0.75), as shown in Figure 4B. Patients with metastatic or recurrent *PTEN*-loss/mutation squamous cell carcinomas (n = 3) and adenocarcinomas (n = 4) achieved a median OS of 6 months (95% CI, 2–10) and 11.3 months (95% CI, 0–22.6), respectively, similar to the median OS of those with metastatic or recurrent *PTEN*-intact squamous cell carcinoma (n = 21; 7 months, 95% CI, 3.1–10.9; *p* = 0.76) and adenocarcinoma (n = 16; 15.7 months, 95% CI, 7.1–24.3; *p* = 0.79), respectively (see Figure 5A and 5B).

## DISCUSSION

In this study, we identified that patients with previously treated, locally advanced or metastatic cervical cancer harboring mutations in the PI3K/AKT/mTOR pathway achieved meaningful clinical benefit from a number of novel therapeutics administered in a phase I cancer center. Of particular interest were the objective responses, prolonged stable disease and PFS among these patients treated with PI3K/AKT/mTOR pathway targeted agents matching the somatic aberrations in this pathway. We conclude from these observations that targeted therapy among these patients with pathway potentiation is a viable strategy for further development.

Patients with metastatic or recurrent carcinoma of the cervix have limited therapeutic treatment options, particularly those in whom combination chemotherapy (without or with anti-angiogenesis agents) have been administered [11, 29, 30]. The Gynecologic Oncology Group (now NRG Oncology) developed phase II queues to explore chemotherapeutic (GOG 127 series, GOG 128 series) and biological agents (GOG 227 series) in this setting. Reflecting the poor anticipated outcomes in these patient cohorts, the statistical decision rules were powered to evaluate “inactive” therapy at ≤ 15% for chemotherapy and ≤ 10% response and/or ≤ 10% non-progression at 6 months, respectively. This provides context upon which to examine the findings in this study.

Several observations are of note. In general, patients with metastatic or recurrent squamous cell carcinoma of the cervix were significantly younger, and had a higher prevalence of *PIK3CA* mutations. However, they displayed lower antitumor activity to currently available phase I clinical trials at MD Anderson associated with significantly shorter OS than patients with metastatic or recurrent adenocarcinoma of the cervix. Second, matched therapy targeting the activated PI3K/AKT/mTOR pathway in patients with metastatic or recurrent squamous cell carcinoma of the cervix led to a favorably higher rate of SD ≥ 6 months/CR/PR as well as significantly longer PFS and OS than non-matched therapy. However, in patients with metastatic or recurrent adenocarcinoma of the cervix, there was no significant difference in OS associated with matched therapy in spite of a higher rate of SD ≥ 6 months/CR/PR and significantly longer PFS, which might be owing to at least three potential factors: lower prevalence of the activated PI3K/AKT/mTOR pathway, intrinsic sensitivity to novel phase I trial therapy available at MD Anderson phase I service, and the presence of *PIK3CA* mutation and/or *PTEN* loss/mutation that cannot stratify for aggressiveness of disease. Third, *PIK3CA* mutation, but not *PTEN* loss/mutation, was associated with significantly longer OS, indicating the differential effects of these genetic aberrations on sensitivity to matched therapy, as well as matched patients and the preferential choices of the treating physicians in the Phase I Clinical Trials Program, MD Anderson for assigning patients to matched therapy.

Of 136 patients with *PIK3CA*-mutation and/or *PTEN*-loss/mutation advanced solid tumors seen in our phase I clinic, 25% achieved SD ≥ 6 months/CR/PR (95%CI, 0.18–0.33) after receiving matched therapy targeting the activated PI3K/AKT/mTOR pathway, and a median PFS of 2.5 months (95% CI, 1.8–3.2) [31]. These outcomes were significantly lower than those reported in the current study of patients with *PIK3CA*-mutation and/or *PTEN*-loss/mutation metastatic or recurrent carcinoma receiving matched therapy. Several confounding factors might have contributed to these differences. Almost half of the patients with metastatic or recurrent cervical carcinoma, especially those with squamous cell carcinoma, presented with *PIK3CA* mutation and/or *PTEN* loss/mutation, and this proportion was significantly higher in patients with advanced solid tumors (48% versus 22%, *p* = 0.002), suggesting that the activated PI3K/AKT/mTOR pathway is a driving mechanism for the survival of cervical carcinoma cells. Another factor is that concurrent mutations or specific molecular profiles might be more important than a single gene mutation. *PIK3CA* mutation was more prevalent in patients with *KRAS* mutation than in patients with wild-type *KRAS* [31], whereas in this report, a low frequency of *KRAS* mutation was found in metastatic or recurrent cervical carcinomas, and in squamous cell carcinomas specifically, no *KRAS* mutation was identified in any of the 26 tested patients (Table 1). Since *PIK3CA* mutation and/or *PTEN* loss/mutation with simultaneous *KRAS* mutation were associated with significantly lower antitumor activity and shorter PFS than *PIK3CA* mutation and/or *PTEN* loss/mutation without simultaneous *KRAS* mutation, two or more coexisting mutations constitute different classes of mutation profiles that predict responses to various biologically targeting agents and/or their combinations and render matched therapy more complicated than expected. When developing efficacious regimens to target the activated PI3K/AKT/mTOR pathway, metastatic or recurrent squamous cell carcinomas of the cervix might be an appropriate clinical model to be tested in early-phase clinical trials because of their high prevalence of *PIK3CA* mutation and/or *PTEN* loss/mutation as previously reported [15, 32–35] and their lack of simultaneous *KRAS* mutation, in agreement with the hypothesis that coexisting *KRAS* mutation become resistant to regimens targeting the activated PI3K/AKT/mTOR pathway.

When considering the clinical relevance of our findings, several limitations should be kept in mind. First, matched therapy includes not only phase I clinical trials of a single agent targeting the activated PI3K/AKT/mTOR pathway, but also phase I clinical trials of an agent targeting this pathway in combination with other biologically targeted agents or conventional chemotherapeutic agents. The importance of using an agent to target the activated PI3K/AKT/mTOR pathway might be overestimated since tumor control might be induced by simultaneous inhibition of other key targets and/or processes by the combination regimens. Second, patients with poor clinical outcomes may have been selectively excluded from being referred to a phase I trial because of rapid tumor progression, poor performance status, insufficient organ function, severe comorbidity, economic issues, and inaccessibility of treatment. Third, molecular studies of mutation profiles were usually conducted on archival tumor specimens whenever available, regardless of whether the specimens had been treated in a phase I clinical trial. Finally, we had a limited sample size available for subgroup analyses, which confounded the ability to validate statistical significance in category assessment. Therefore, conclusions from this retrospective study should be considered preliminary evidence to generate hypotheses, which require further validation in larger prospective studies.

In conclusion, our results showed that almost half of the patients with metastatic or recurrent squamous cell carcinoma of the cervix had *PIK3CA* mutation and/or *PTEN* loss/mutation without coexisting *KRAS* mutation, providing an appropriate patient population to the test efficacy of a regimen including a single agent targeting the PI3K/AKT/mTOR pathway or a combination regimen with either another biologically targeted agent and/or conventional chemotherapeutic agent. Matched therapy targeting a specific mutation has provided apparent clinical benefits for cancer patients. Complicated mutation profiles might be more important and useful than a single gene mutation in predicting antitumor activity and clinical efficacy to a specific regimen [36–40]. Therefore, future development and evaluation of novel regimens targeting the activated PI3K/AKT/mTOR pathway for the purpose of translating high antitumor activity to prolonged survival benefit is warranted in larger prospective clinical trials for the treatment cervical carcinoma, especially squamous cell carcinoma.
